# Halogenated hydrocarbon solvent-related cholangiocarcinoma risk: biliary excretion of glutathione conjugates of 1,2-dichloropropane evidenced by untargeted metabolomics analysis

**DOI:** 10.1038/srep24586

**Published:** 2016-04-18

**Authors:** Yu Toyoda, Tappei Takada, Hiroshi Suzuki

**Affiliations:** 1 Department of Pharmacy, The University of Tokyo Hospital, 7-3-1 Hongo, Bunkyo-ku, Tokyo 113-8655, Japan

## Abstract

Recently, the International Agency for Research on Cancer issued a warning about the carcinogenicity of 1,2-dichloropropane (1,2-DCP) to humans based on an epidemiological study suggesting a relationship between the incidence of cholangiocarcinoma and occupational exposure to halogenated hydrocarbon solvent comprised mostly of 1,2-DCP. Although this dihaloalkane has been used in various industrial fields, there has been no biological evidence explaining the cholangiocarcinoma latency, as well as little understanding of general cholangiocarcinoma risk. In the present study, we explored the biliary excretion of 1,2-DCP metabolites by an untargeted metabolomics approach and the related molecular mechanism with *in vitro* and *in vivo* experiments. We hypothesized that the biliary excretion of carcinogens derived from 1,2-DCP contribute to the increased cholangiocarcinoma risk. We found that 1,2-DCP was conjugated with glutathione in the liver, and that the glutathione-conjugated forms of 1,2-DCP, including a potential carcinogen that contains a chloride atom, were excreted into bile by the bile canalicular membrane transporter, ABCC2. These results may reflect a risk in the backfiring of biliary excretion as a connatural detoxification systems for xenobiotics. Our findings would contribute to uncover the latent mechanism by which the chronic exposure to 1,2-DCP increases cholangiocarcinoma risk and future understanding of cholangiocarcinoma biology.

Halogenated solvents are volatile organic chemicals consisting of one hydrocarbon or a short-chain of hydrocarbons that includes one or more chlorine or bromine atoms. Most of these chemicals have been used as degreasers and solvents for various industrial products^1^. For workers involved in the production or use of halogenated solvents, there is a risk of solvent exposure by inhalation or by dermal contact. Since some of halogenated hydrocarbons and/or their metabolites are recognized as toxic substances. Industrial use of such chemicals is restricted by national authorities in developed countries.

In 2014, the International Agency for Research on Cancer (IARC) has re-classified 1,2-dichloropropane (DCP) into Group 1 (Carcinogenic to humans) from Group 3 (Not classifiable as to its carcinogenicity to humans)^2^. This classification change was based on observations that some young employees who had a long history of exposure to halogenated hydrocarbon solvents composed mostly of 1,2-DCP developed occupational cholangiocarcinoma (bile duct cancer)^3^. Actually, it was recognized as a big social problem in Japan that an outbreak of cholangiocarcinoma among young workers at 25–45 years old with long-term exposure to very high levels of 1,2-DCP at printing plants where 1,2-DCP was used as an ink-removal agent^2^,^3^,^4^. Furthermore, 1,2-DCP is a synthetic chlorinated solvent and is obtained as a by-product of the production of epichlorohydrin, which is used as a major chemical building block. 1,2-DCP has been also used as a chemical intermediate in the production of some other organic chemicals^2^. Therefore, to improve occupational safety by more appropriate regulations, it is important to understand the latent mechanisms of biliary carcinogenesis related to 1,2-DCP.

The most serious problems associated with the inhalation of 1,2-DCP include the development of cholangiocarcinoma, an epithelial cell malignancy arising from the biliary-duct system^4^. This cancer is classified into intrahepatic and perihilar or distal extrahepatic types according to its anatomic location. Most of these cancers arise *de novo*. The incidence of cholangiocarcinoma has been increasing in developed countries^4^,^5^,^6^. Therapies for the treatment of cholangiocarcinoma are less effective than for other cancers. This is because the molecular pathogenesis of cholangiocarcinoma is poorly understood^4^,^6^. In addition, few risk factors for cholangiocarcinoma have been identified, with the exceptions of liver fluke infection, hepatitis, and sclerosing cholangitis^4^. Therefore, further understanding of cholangiocarcinoma latency and its biology is needed.

There has been no biological evidence explaining the latent association between exposure to 1,2-DCP and the pathogenesis of cholangiocarcinoma. On the other hand, some *in vivo* experiments showed that 1,2-DCP-exposed mice developed malignant lung and hepatocellular tumours, suggesting the carcinogenicity of 1,2-DCP and/or its metabolites^7^,^8^. In the liver, a major organ in the metabolism of various compounds, xenobiotics are generally converted to the hydrophilic form by Phase I (oxidation) and Phase II (conjugation) reactions, and are subsequently excreted into the extracellular space by the Phase III (elimination) system^9^ such as ATP-binding cassette sub-family C member 2 (ABCC2) and ABCG2. These efflux transporters are located on the bile canalicular membrane of hepatocytes^10^. Although biliary excretion of 1,2-DCP has never been reported, both depletion of hepatic GSH^11^ and urinary excretion of mercapturate form of 1,2-DCP (an ultimate metabolite of GSH-conjugates)^12^,^13^ are observed in 1,2-DCP-administered rodents, supporting that GSH is conjugated with 1,2-DCP. In this context, we hypothesized that 1,2-DCP would be excreted into bile as its GSH-conjugated form.

We hypothesized that the biliary excretion of some carcinogens derived from the halogenated hydrocarbon 1,2-DCP will increase the cholangiocarcinoma risk. Because the concentration of compounds excreted into bile are generally higher than blood concentrations, our hypothesis would be plausible for bile duct-specific carcinogenesis in 1,2-DCP-related occupational cholangiocarcinoma cases in humans^3^. In addition, our experiences addressing the physiological and clinical importance of bile canalicular transporters^14^,^15^ and the detoxification potential of the liver^16^ strongly suggested to us that the conjugated form of 1,2-DCP was likely to be excreted into bile by Phase III machinery according to the connatural detoxification system in the body.

In the present study, we focused on the biliary excretion of 1,2-DCP metabolites and related mechanisms. First, we determined the biliary-excreted metabolites of 1,2-DCP using an untargeted metabolomics approach followed by differential analysis. We next showed that GSH-conjugated forms of 1,2-DCP were excreted into bile by ABCC2. This biliary secretion of potential carcinogens was also confirmed in humanized-liver mice, supporting the extrapolation of our results to humans. In addition, in order to identify the candidates for biomarkers related to the exposure to 1,2-DCP, we analysed the metabolic changes in serum.

## Results

### GSH-conjugated DCP metabolites (GS-DCPs) were markedly excreted into bile in 1,2-DCP administered rodents

In order to test our hypothesis that 1,2-DCP and/or its metabolites are excreted into bile, we performed untargeted metabolomics and differential analyses. Four hours after the oral administration of 1,2-DCP (500 mg/kg) to some rodents (C57BL/6J mice, FVB/NJcl mice, and Sprague Dawley (SD) rats), bile specimens of each rodent were collected by bile cannulation. The dosage amount of 1,2-DCP in the present study was comparable to the estimated-intake amount of 1,2-DCP in the occupational cholangiocarcinoma cases: 4.6–31 g/day^17^, which is consistent with 76–513 mg/kg/day in normal-body weight (60.4 kg) men. Specimens were analysed by Orbitrap UPLC-MS/MS system, as described in details in the *Methods*. In order to search for metabolites derived from 1,2-DCP, we explored and selected some MS peaks with a significantly higher intensity in the 1,2-DCP-administered group than the control group. Considering the previous studies^12^,^13^ that have reported the production of the mercapturate form of 1,2-DCP *in vivo*, we further utilized the monoisotopic peak information ^34^S (derived from GSH) and ^37^Cl (derived from 1,2-DCP) as markers for judging on whether the peaks of interest were derived from 1,2-DCP-metabolites.

**Figure 1:**
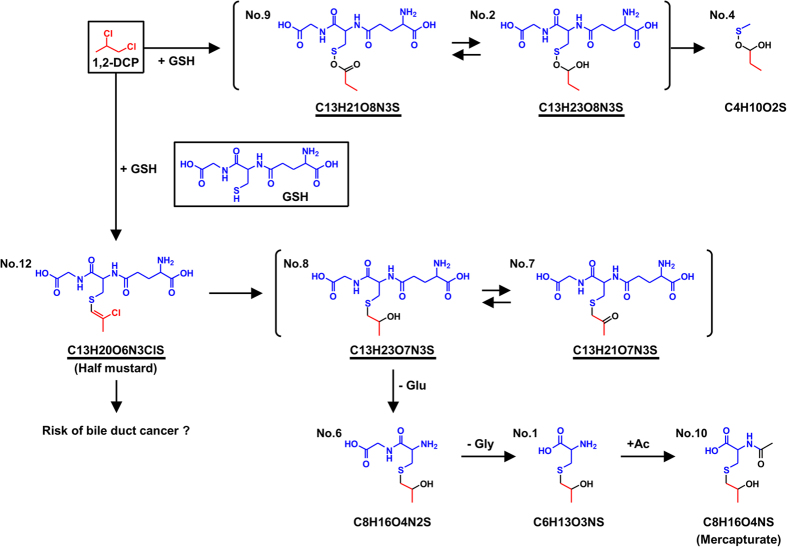
Proposed metabolic pathways of 1,2-DCP in the liver-biliary system. As a result of untargeted metabolomics approach followed by differential analyses, 13 peaks of interest were selected (summarized in Supplementary Table S1). Based on the results of LC-MS/MS analysis (accurate mass, isotopic pattern, and fragment ions for target metabolites), we determined the putative chemical structures for each peak. According to the structural information, we finally selected nine compounds as the metabolites of 1,2-DCP. No. 12 and No. 10 are correspond to a half mustard and mercapturate form, respectively. The presence of chloride ion in the metabolite No. 12 was confirmed by the isotopic peak derived from ^37^Cl. Underlined compounds are GS-DCPs and include glutathione-derived chemical structure.

We identified 13 peaks as candidates for 1,2-DCP metabolites in bile (Supplementary Table S1). The parent compound (1,2-DCP) was not detected in the bile samples of 1,2-DCP-administered rodents. For further analyses of their chemical structures, we acquired the MS/MS data corresponding to each peak. Based on the information about composition formula calculated by accurate mass and fragmentation patterns identified by MS/MS analysis, we determined the putative chemical structures for each peak. According to the structural information, we finally selected nine compounds as the metabolites of 1,2-DCP (Fig. 1). Interestingly, metabolite No. 12 contained both one chlorine and one GSH in its chemical structure. This means that 1,2-DCP should be conjugated with GSH and excreted into bile. Considering the conformational change and conjugation pattern between the 1,2-DCP-derived carbon skeleton and GSH, two metabolic pathways were proposed as described in Fig. 1.

### Biliary excretion of GS-DCPs was decreased in *Abcc2*-deficient model rats (EHBRs), but not in *Abcg2* KO mice

**Figure 2:**
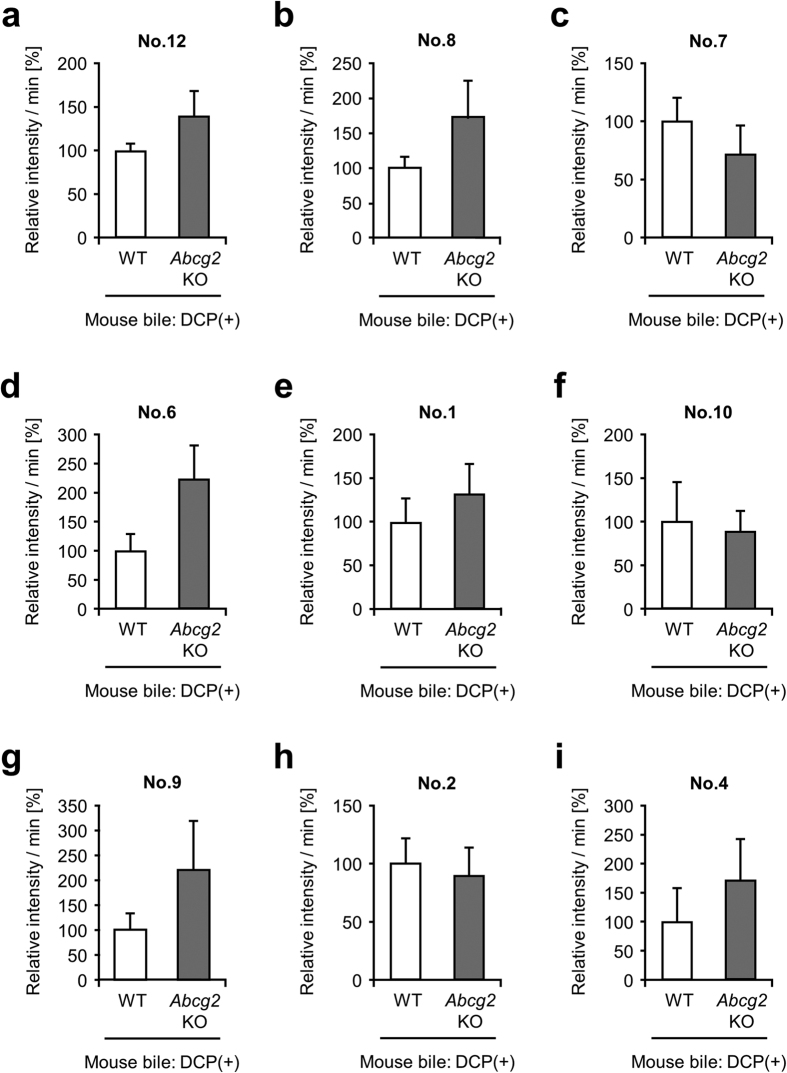
Comparison of biliary levels of each 1,2-DCP metabolite between 1,2-DCP-administered WT and *Abcg2* KO mice. Bile specimens of 1,2-DCP-administered WT and *Abcg2* KO mice were analysed using LC-MS/MS. Relative intensity was calculated as the ratio of each metabolite level in the *Abcg2* KO group to that of the WT group. This value is expressed as the mean ± S.E.M. *n* = 6 (WT), 7 (*Abcg2* KO). Statistical analyses for significant differences were performed according to parametric, Student’s *t* test. There was no significant difference in the biliary level of each 1,2-DCP metabolite between WT and *Abcg2* KO mice.

In order to gain insight into the molecular mechanisms involved in the biliary excretion of GSH-conjugated 1,2-DCPs (GS-DCPs; No. 12, No. 8, No. 7, No. 9 and No. 2 in Fig. 1) and other related metabolites, we further conducted metabolomics analyses by using *Abcg2* knockout (KO) mice and Eisai Hyper-bilirubinemia Rats (EHBR is an *Abcc2-*deficient animal model), because both two ABC transporters play a pivotal role in ATP-dependent biliary excretion of conjugated xenobiotics as components of Phase III machinery. First, to examine the effect of Abcg2 on the biliary excretion of GS-DCPs, we semi-quantitatively compared the biliary levels of each 1,2-DCP metabolite between 1,2-DCP administered-WT mice and -*Abcg2* KO mice. There was no significant difference in the biliary levels of each metabolite between the two groups (Fig. 2). This result suggests that Abcg2 would not be responsible for the biliary excretion of 1,2-DCP metabolites including GS-DCPs.

**Figure 3:**
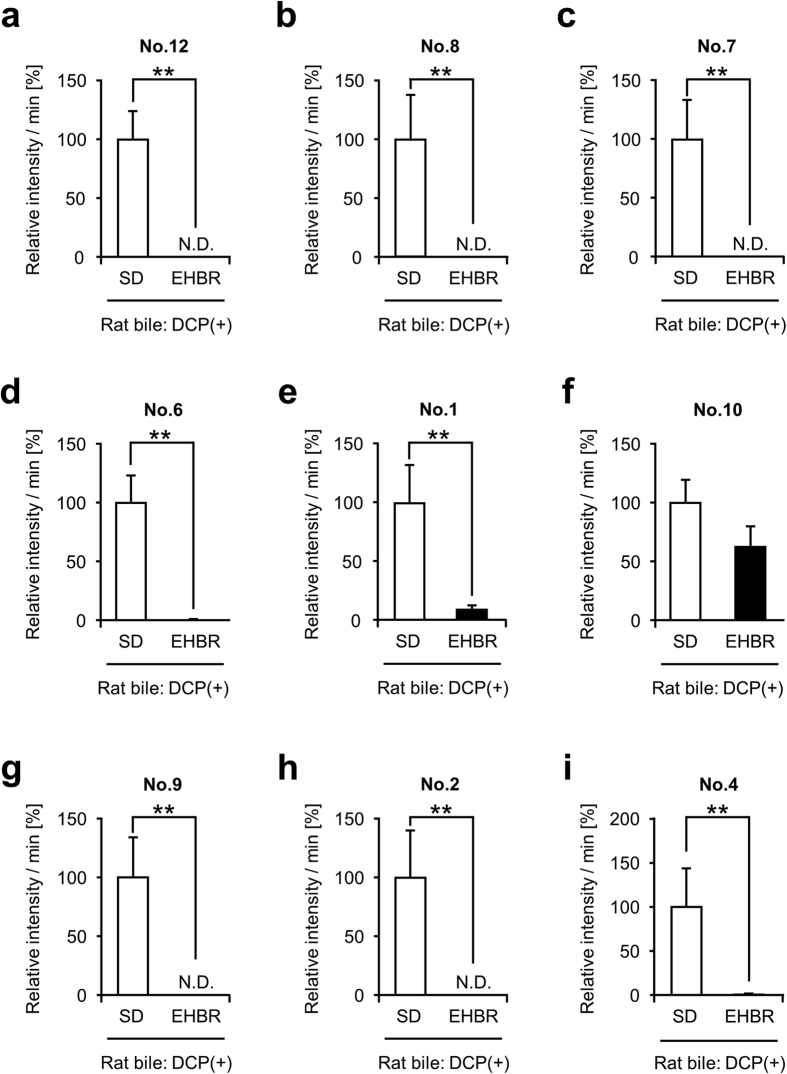
Comparison of biliary levels of each 1,2-DCP metabolite between 1,2-DCP-administered SD rats and *Abcc2*-deficient EHBRs. Bile specimens of 1,2-DCP-administered SD rats and *Abcc2*-deficient EHBRs were analysed using LC-MS/MS. Relative intensity was calculated as the ratio of each metabolite level of 1,2-DCP in the EHBR group to that in the SD rat group. This value is expressed as the mean ± S.E.M. *n* = 9 (SD rat), 10 (EHBR). Statistical analyses for significant differences were performed according to a parametric Student’s *t* test or a nonparametric Mann-Whitney *U* test (**P < 0.01). N.D. not detected.

**Figure 4:**
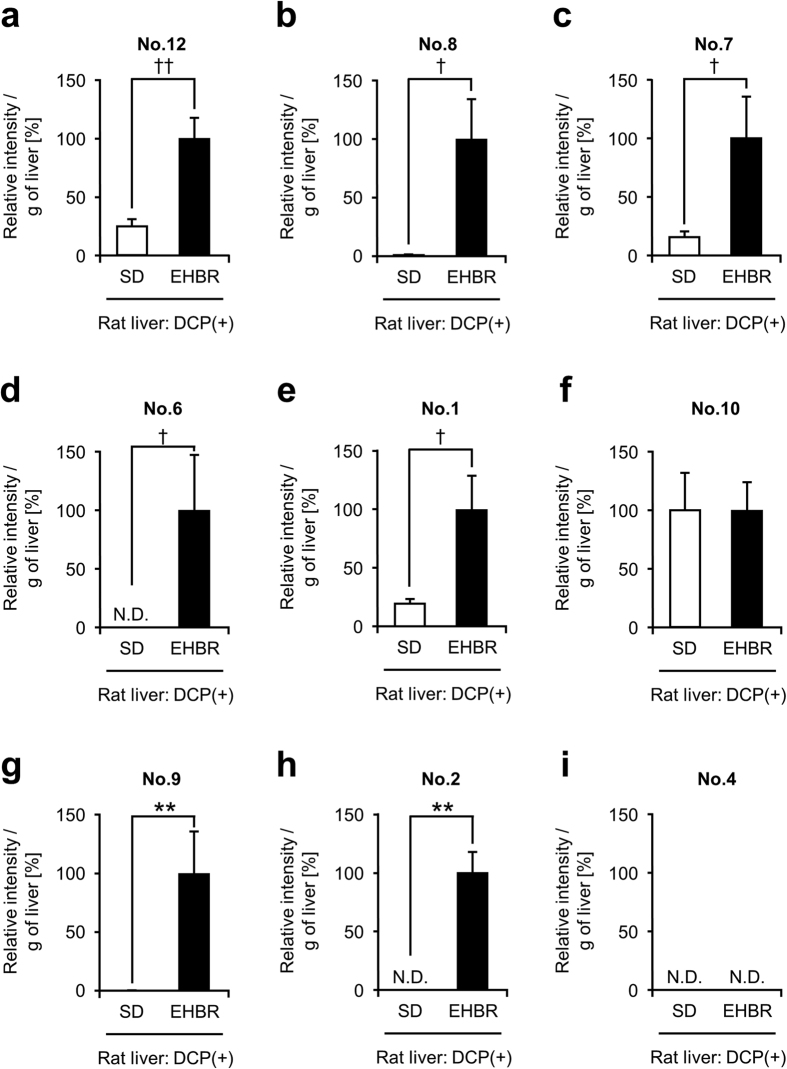
Comparison of liver levels of each 1,2-DCP metabolite between 1,2-DCP-administered SD rats and *Abcc2*-deficient EHBRs. Liver tissues of 1,2-DCP-administered SD rats and *Abcc2*-deficient EHBRs were analysed using LC-MS/MS. Relative intensity was calculated as the ratio of each metabolite level in the SD rat group to that in the EHBR group. This value is expressed as the mean ± S.E.M. *n* = 6. Statistical analyses for significant differences were performed according to a parametric Student’s *t* test (^†^*P* < 0.05; ^††^*P* < 0.01) or a nonparametric Mann-Whitney *U* test (**P < 0.01). N.D. not detected.

Next, focusing Abcc2, we conducted the same experiment by using SD rats (WT) and EHBRs. The biliary levels of all metabolites in EHBRs in Fig. 1, except for No. 10, were significantly lower than those in SD rats (Fig. 3). This suggested contribution of Abcc2 to the biliary excretion of GS-DCPs. We subsequently examined the hepatic levels of each metabolite (Fig. 4). Contrary to the lower biliary levels of each metabolite, the hepatic levels of seven metabolites (No. 12, No. 8, No. 7, No. 6, No. 1, No. 9, and No. 2) in EHBRs were significantly higher than that in SD rats. Remarkably, the biliary/hepatic levels of most of GS-DCPs in EHBRs were much lower than those in SD rats (Supplementary Fig. S1). These results strongly suggest that Abcc2 is responsible for the excretion of GS-DCP from liver to bile. This is also consistent with the well-known fact that ABCC2 mediates the biliary excretion of GSH and GSH-related compounds^18^.

### Spontaneous reaction of GSH with 1,2-DCP resulted in GS-DCP production

We next examined whether the GSH-conjugation with 1,2-DCP spontaneously progressed or not, since in some cases GSH reacts with electrophilic chemicals by a non-catalytic process as well as by a catalytic process^19^,^20^. Interestingly, the simple incubation of 1,2-DCP and GSH in potassium phosphate buffer resulted in the spontaneous production of the GS-DCPs (Supplementary Fig. S2). Notably, the reaction temperature-dependent increase of No. 12 levels in the incubation mixture was correlated with the decrease of GSH. These results suggest that at least No. 12 (half mustard form) could be produced by non-catalytic process. Accordingly, we could obtain GS-DCPs-enriched fractions by separation of the reaction mixture (Supplementary Fig. S3).

### ABCC2 did directly transport GS-DCPs by an ATP-dependent manner

**Figure 5:**
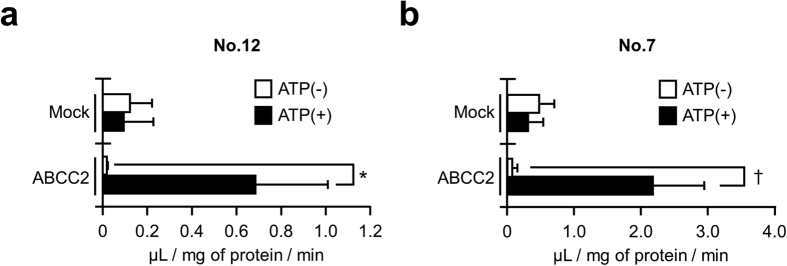
ABCC2-mediated transport of GSH-conjugated 1,2-DCP metabolites. Transport of GS-DCPs was examined with human ABCC2-expressing vesicles. Incorporation activities of No. 12 (**a**) and No. 7 (**b**) in mock and ABCC2-expressing vesicles were shown with or without ATP. The incubation condition was 37 °C for 10 min. ATP-dependent transport was calculated by subtracting the transport activity in the presence of 5 mM AMP (ATP (−)) from the activity in the presence of 5 mM ATP. Data are expressed as the mean ± S.D. *n* = 3. Statistical analyses for significant differences were performed according to a parametric Student’s *t* test (^†^*P* < 0.05) or a nonparametric Mann-Whitney *U* test (*P < 0.05).

Since the biliary excretion of GS-DCPs in EHBRs lacking functional Abcc2 was much lower than that in SD rats, we supposed that GS-DCPs should be excreted into bile by Abcc2. To examine whether GS-DCPs are substrates of ABCC2, we performed an *in vitro* transport assay by using ABCC2-expressing plasma membrane vesicles for two GS-DCPs that we had adequately obtained. As expected, ATP-dependent incorporation of GS-DCPs was detected in ABCC2-expressing vesicles but not in control vesicles (Fig. 5). The ATP-dependent transport activities of GS-DCPs in ABCC2-expressing vesicles were 0.67 ± 0.33 μL/mg of protein/min (for No. 12) and 2.11 ± 0.70 μL/mg of protein/min (for No. 7). In contrast, transport in mock vesicles was almost zero. Taken together with the *in vivo* experiments using EHBRs, our data indicate that most of GS-DCPs were excreted into bile by Abcc2.

### GS-DCPs were excreted into bile in humanized-liver mice

**Figure 6:**
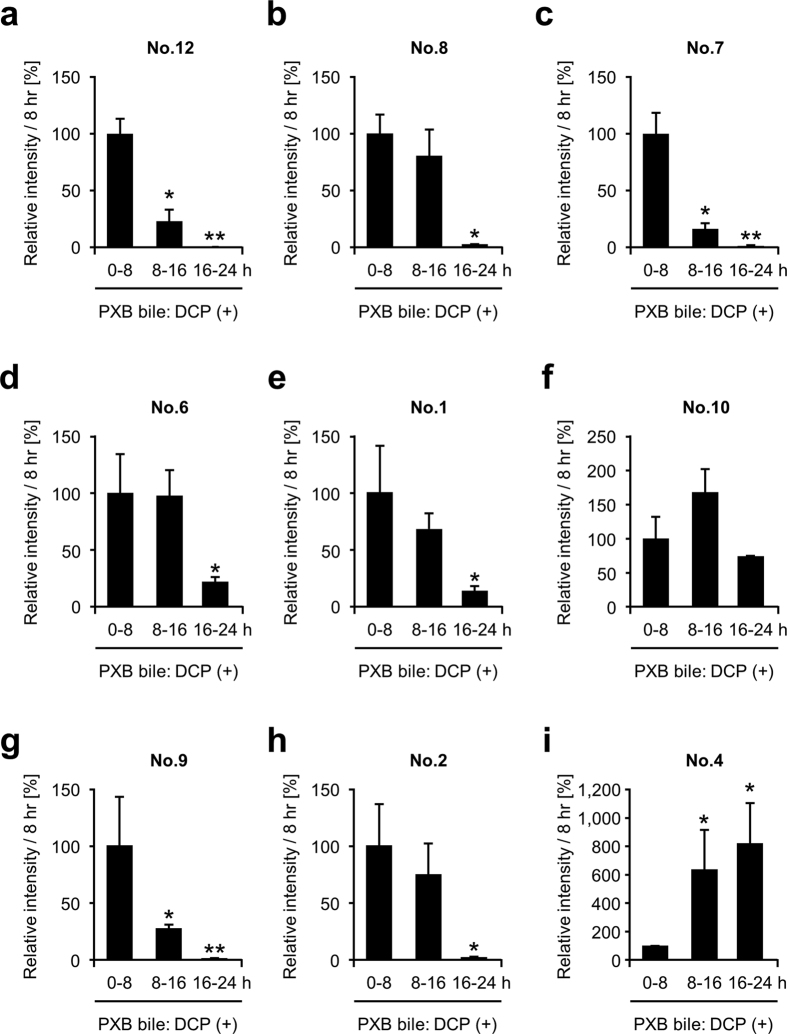
Time course of biliary levels of each 1,2-DCP metabolite in 1,2-DCP-administered humanized-liver mice. Bile specimens of 1,2-DCP-administered PXB mice were collected for 24 hours every 8 hours, and analysed using LC-MS/MS. The biliary levels of each metabolite at 0–8 h is defined as percent control (100%). Data are expressed as the mean ± S.E.M. *n* = 3. Statistical analyses for significant differences were performed according to Bartlett’s test, followed by Shirley-Williams’s multiple-comparison test (*P < 0.05; **P < 0.01 vs. control).

In order to extend our above understanding to humans, we further examined the biliary excretion of DCP metabolites in humanized-liver mice known as PXB mice^21^. Since the livers of PXB mice are replaced by human hepatocytes, these mice have the human hepatic ability to metabolize and excrete xenobiotic compounds. Before the use, we confirmed the liver humanization by the presence of human albumin (11.9 ± 0.8 mg/mL) in the blood of PXB mice which were generated using human hepatocytes derived from a single donor. Immediately after 1,2-DCP administration, bile samples of PXB mice were collected by cannulation and pooled for 24 hours per 8 hours. Bile analysis revealed that DCP metabolites including GS-DCPs, were excreted into bile (Fig. 6). This result suggests that 1,2-DCP could be metabolized and excreted into bile in humans exposed to 1,2-DCP: a similar effect was observed in rodent models examined in the present study.

Biliary levels of GS-DCPs (No. 12, No. 8, No. 7, No. 9, and No. 2), No. 6, and No. 1 in 1,2-DCP-administered PXB mice decreased in a time-dependent manner, while those of No. 10 and No. 4 exhibited different patterns (Fig. 6). Considering that No. 10 and No. 4 are suggested to be the ultimate metabolites according to their putative structure (Fig. 1), these different patterns may reflect the time-dependent decrease of intermediate metabolites and increase of ultimate metabolites in bile. In this context, these results also support the putative metabolic pathway of 1,2-DCP described in Fig. 1.

### Detection of biomarker candidates in serum

In order to examine whether the biliary excretion of DCP metabolites could be assessed by using blood specimens, we investigated the metabolic change in serum under the 1,2-DCP-administered condition in SD rats. Serum levels of DCP metabolites were analysed by LC-MS/MS system. Although No. 6 and No. 2 were not detected, the other 7 metabolites were detected in the serum of 1,2-DCP-administered SD rats (Table 1). Hence, a plausible explanation would be that the accumulated metabolites of 1,2-DCP were, at least partially excreted from the liver to blood in addition to biliary excretion.

We further compared the metabolite levels in serum, bile, and liver in SD rats (Table 1). As a result, serum levels of GS-DCPs (No. 12, No. 8, No. 7, No. 9, and No. 2), No. 6, and No. 1 were much lower than their biliary levels, as well as their hepatic levels. In contrast, serum and biliary levels of No. 10 were not significantly different: 2.98 ± 0.69 vs. 3.80 ± 0.42 (intensity/mL), respectively. In addition, normalization of the relative levels of each metabolite to No. 10 levels in serum, bile, and liver (Supplementary Fig. S4) suggested that No. 10, as well as No. 1 and No. 4, tend to distribute to blood compared with other 1,2-DCP metabolites. Hence, these metabolites would be good serum biomarker candidates.

## Discussion

In the present study, we analysed the metabolic conversion of 1,2-DCP *in vivo* and its biliary excretion in order to uncover the latent relationship between the risk of cholangiocarcinoma and the excess exposure to 1,2-DCP. When administered to rodents, 1,2-DCP was excreted into bile in GSH-conjugated forms (Fig. 1). Significant decreases in the biliary levels of GS-DCPs in EHBRs lacking Abcc2 function indicate that Abcc2 is the transporter most responsible for biliary excretion of GS-DCPs (Fig. 3). This is also consistent with direct transport of GS-DCPs by ABCC2 (Fig. 5). Furthermore, the biliary excretion of GS-DCPs was observed in humanized-liver mice (Fig. 6), strongly suggesting the possibility of this phenomenon in humans. Hence, in cholangiocarcinoma patients who had a long duration of exposure to 1,2-DCP, GS-DCPs would be continuously excreted into bile. Considering the tissue selectivity of 1,2-DCP-related to cancer risk in humans, this biliary excretion of 1,2-DCP metabolites would be responsible for the cholangiocarcinoma.

How would the biliary excretion of GS-DCPs be related to the cholangiocarcinoma risk? In general, glutathione conjugation (a Phase II reaction) and the subsequent export of conjugated metabolites mediated by the GS-X pump (a Phase III reaction) play a pivotal role in the biological inactivation of xenobiotics and their elimination^9^. However, it has been also pointed out that in some cases, Phase II reactions result in the formation of genotoxic electrophiles. In fact, Ozawa and Guengerich reported that 1,2-dibromoethanes, members of the dihaloalkanes class of compounds, were activated by GSH conjugation to genotoxic electrophiles such as episulfonium ion, resulting in the formation of the DNA-adduct (S-[2-N7-guanyl]ethyl)GSH)^22^. To date, several lines of evidence have indicated the mutagenic and carcinogenic potential of 1,2-dihaloalkanes due to formation of their reactive intermediates via glutathione conjugation^23^,^24^. During this process, a residual halogen atom is essential for the formation of episulfonium ion. In this context, our finding that GS-DCPs including No. 12 with a chlorine atom, were highly excreted into bile would be the plausible explanation for cholangiocarcinoma risk by 1,2-DCP.

In addition, a long duration of exposure to dihaloalkanes such as dichloromethane, 1,2-dichloroethane and 1,2-DCP in rodents reportedly resulted in the liver-hepatocellular carcinoma^8^,^25^, supporting carcinogenic properties of 1,2-DCP. However, the reason why hepatocarcinoma but not cholangiocarcinoma was induced in the 1,2-DCP administered rodents remains unclear. One possibility is that rodents may be more susceptible to hepatocarcinoma compared with cholangiocarcinoma and that in humans the opposite may be the case. Species difference between rodents and humans might be supported by following examples. Some xenobiotics, such as fibrate drugs, cause liver cancers when chronically administered to rodents, while humans have no risk of cancer by fibrates^26^,^27^. In addition, no spontaneous cases of cholangiocarcinoma have been reported in rodents, except for in chemically-induced^28^,^29^ or genetically-engineered^30^,^31^ experimental models. Hence, a subject for future investigations is to analyse clinical samples from the occupational cholangiocarcinoma cases^3^. Indeed, we have already indicated that potentially carcinogenic compounds might be excreted into bile in humans as well as rodents, as indicated by humanized-liver mice experiments (Fig. 6). The potentially carcinogenic compound, No. 12, and/or the GS-DCP-DNA adduct would be detected in the bile duct cancer tissue in humans.

Our finding that metabolites of 1,2-DCP were excreted into bile sheds light on an overlooked-elimination route of this dihaloalkane. Previous studies demonstrated that after oral or inhalation exposure, [^14^C]1,2-DCP (^14^C activity) was detected in faeces (about 6–8% of dose) regardless of exposure route in rodents, although the principal routes of elimination of 1,2-DCP were urine and expired air^32^,^33^. In addition, previous studies also reported that no parent 1,2-DCP was observed in any urine samples^12^,^32^,^33^, suggesting the rapid conversion of 1,2-DCP to its metabolites *in vivo*. Considering that 1,2-DCP administered by inhalation would not directly distribute to intestine, the faecal excretion of ^14^C implied the biliary route for elimination of 1,2-DCP and/or its metabolites, which is consistent with the results of the present study. Thus, 1,2-DCP metabolite(s) excreted into bile may affect human health, even if the contribution of biliary excretion to total bodily clearance for 1,2-DCP is small.

We have succeeded in the identification of GS-DCPs for the first time (Fig. 1). Based on the presence of 1,2-DCP-derived mercaputurate in urine, some researchers deduced that 1,2-DCP was conjugated with GSH in its metabolic pathway^12^,^32^,^33^. There had been, however, no direct evidence indicating the presence of GS-DCPs. Presently, we have achieved more detailed understanding, although we could not determine the accurate structure of each of the 1,2-DCP metabolites owing to the experimental limitation of obtaining the corresponding standard compounds. In any cases, the metabolic pathway of 1,2-DCP in the liver that we have proposed (Fig. 1) is consistent with both the conversion pathway of mercapturic acid^34^ and the remarkable decrease of hepatic GSH in 1,2-DCP-administered rats^11^ described in previous reports.

We found that the reaction of GSH (6 mM) with 1,2-DCP *in vitro* is non-catalytic (Supplementary Fig. S2). Since GSH levels are 5 to 10 mM in the liver and 1 to 2 mM in many other tissues^20^, the liver would be the most susceptible to spontaneous production of GS-DCPs. However, most dihaloalkanes have been reported to be activated as potentially genotoxic compounds by catalytic GSH conjugation by GSH transferases (GSTs), as well as by cytochrome P450-mediated oxidation^24^. In humans, at least, 16 cytosolic GST subunits exist and function as their dimers^35^. Since mammalian theta class GSH transferases (GSTTs) catalysed the conjugation of GSH with haloalkanes including 1,2-dihaloethanes and dihalomethanes^36^,^37^, GSTTs may catalyse GSH conjugation of 1,2-DCP *in vivo*. However, the involvement of a specific GST in that reaction remains unknown. Therefore, whether specific GST could facilitate the production of the half mustard form of 1,2-DCP (GS-DCP-Cl, No. 12) need clarification. Interestingly, accumulating evidence suggests the influence of GST polymorphisms in cancer susceptibility^38^. In humans, *GSTT1* and *GSTM1* genotypes are likely to correlate with hepatic cancer risks^39^, and a positive association between GSTO1*A140D and cholangiocarcinoma risk had been reported^40^. Combined with the high allele frequency in worldwide populations of *GSTT1* and *GSTM1 null* genotypes^38^, further studies focusing on the GSTs should contribute to a better understanding of genetic risk factors for cholangiocarcinoma. In addition, an association of genetic variation in *ABCC2* with susceptibility to bile duct cancer has been pointed out^41^, which would warrant further investigations for cholangiocarcinoma risk focusing on biliary excretion system as well as hepatic metabolizing system.

**Figure 7:**
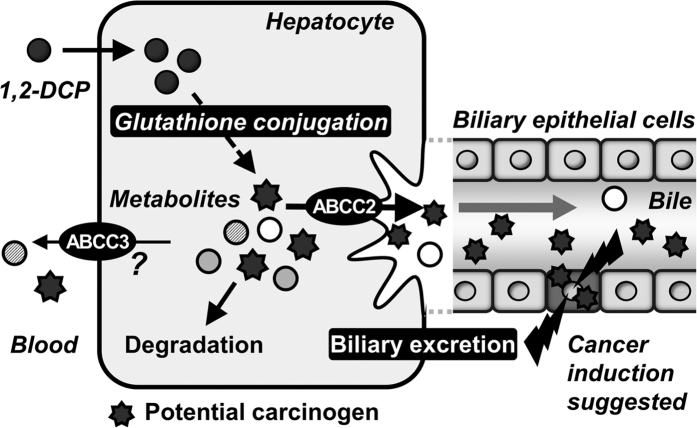
Schematic illustration of metabolism and elimination processes of 1,2-DCP and its metabolites in liver-biliary system. In the liver, 1,2-DCP is metabolized to GS-DCPs via the reaction with GSH, followed by excretion of most glutathione conjugates into bile by ABCC2 localized on the bile canalicular membrane of hepatocytes. Harmful effect on biliary epithelial cells probably cause cholangiocarcinoma. However, a portion of 1,2-DCP metabolites are secreted into blood from the liver. ABCC3 would be involved in this process.

In the present study, we focused on ABCC2 and ABCG2 as a typical Phase III machinery in the liver. These efflux pumps transport vast and chemically diverse substrates, including conjugated organic anions, across the plasma membrane at the cost of ATP hydrolysis^10^. ABCC2 is mainly able to transport GSH-, glucuronide-, and sulphate-conjugated organic anions, whereas ABCG2 has a preference for sulphate conjugates of steroids and xenobiotics compared to GSH- or glucuronide-conjugated metabolites^42^. This distinguishing feature in the substrate specificities of ABCC2 and ABCG2 is consistent with our results demonstrating that the biliary excretion of GS-DCPs was mostly disappeared in *Abcc2*-deficient EHBRs (Fig. 3), but not in *Abcg2* KO mice (Fig. 2). Considering the direct transport of GS-DCPs by ABCC2 (Fig. 5), the metabolism and biliary excretion of 1,2-DCP in the liver are summarized as described in Fig. 7. In fact, 1,2-DCP would be activated as a half mustard form (GS-DCP-Cl, No. 12) by spontaneous and/or catalytic GSH conjugation (Phase II system) in the liver. Then, the metabolite would be excreted into bile via a bile canalicular transporter ABCC2 (Phase III system) and may affect biliary epithelial cells. These mechanisms would be the plausible explanation for the cause of 1,2-DCP-related cholangiocarcinoma, especially in extrahepatic cases that are proposed to arise from the biliary epithelium and peribiliary gland^43^.

Furthermore, we also found that some metabolites of 1,2-DCP were detectable in blood, although most of them were mostly excreted into bile (Table 1). It has been reported that some endogenous substrates of ABCC2 are partially secreted from the liver into blood by ABCC3, a member of the ABC transporter family localized on the basolateral membrane of hepatocytes^44^. Considering the similarity of substrate specificity between ABCC2 and ABCC3, ABCC3 is likely to be involved in the secretion of GS-DCPs from liver to blood (Fig. 7). In this context, GS-DCPs might be additional candidates for biomarkers in occupational cholangiocarcinoma as well as No. 10, the mercapturate form of 1,2-DCP.

Our results could propose a new paradigm for understanding the cholangiocarcinoma risk. Some risk factors for cholangiocarcinoma, such as parasitic infections, inflammation caused by hepatic/biliary diseases, and hepatolithiasis characterized by the presence of gallstones have been recognized^43^,^45^,^46^. However, the molecular mechanisms involved in the development of cholangiocarcinoma are poorly understood. In the present study, we have found a potential risk for cholangiocarcinoma caused by the failure of connatural detoxification systems in the body, suggesting the presence of carcinogenic mechanisms related to Phase III system as well as Phase II system.

In conclusion, we revealed that 1,2-DCP is conjugated with GSH, and that its metabolites including GS-DCP-Cl, are excreted into bile by a bile canalicular membrane transporter ABCC2. These findings partially uncover the latent relationship between chronic exposure to 1,2-DCP and cholangiocarcinoma risk in humans, with backfire of the Phase III system increasing the risk of biliary disorders as a plausible explanation. As such, the work reported here utilized the strength of an untargeted metabolomics approach in order to pave the way to further investigation of the risk of biliary disorders related to bile component changes caused by our dwelling/working environments and eating habits.

## Methods

### Materials

The following compounds were purchased from commercial sources indicated in parentheses: 1,2-DCP, ATP, AMP, creatine phosphate disodium salt tetrahydrate and creatine phosphokinase type I from rabbit muscle (Sigma-Aldrich, St. Louis, MO); glutathione reduced form (Nacalai Tesque Inc., Kyoto, Japan); leukotriene C4 (LTC4)-d5 (Cayman Chemical Co., Ann Arbor, MI); 5,5′-dithiobis(2-nitrobenzoic Acid), also called as Ellman’s reagent^47^ and olive oil (Wako Pure Chemical Industries Ltd., Osaka, Japan); 0.1% Formic Acid in Water (v/v), Solvent Blends and 0.1% Formic Acid in Acetonitrile (v/v), Solvent Blends in Optima LC/MS grade (Thermo Fisher Scientific K.K., Yokohama, Japan); ABC Transporter Vesicles for human ABCC2 and control (GenoMembrane Co., Ltd., Yokohama, Japan). All other chemicals used were commercially available and of analytical grade.

### Animals

Wild type C57BL/6J mice and FVB/NJcl mice were purchased from CLEA Japan (Tokyo, Japan). *Abcg2* KO mice (FVB.129P2-Abcg2) were obtained from Taconic Farms (Hudson, NY) and have been bred in our laboratory^48^. SD rats and EHBRs were purchased from Japan SLC, Inc. (Shizuoka, Japan). These animals used in the present study were males at 6–10 weeks of age and were maintained on a standard diet and water *ad libitum* under 12-hour light and dark cycles as described previously^49^. Chimeric mice with humanized liver (PXB mice; PhoenixBio Co., Ltd., Hiroshima, Japan) were generated as previously described^50^. All PXB mice in the present study were transplanted with frozen human hepatocytes derived from a single donor. Male PXB mice at 13–16 weeks of age were used after the confirmation of liver humanization (estimated as 84 ± 2%) by the presence of human albumin (11.9 ± 0.8 mg/mL) in mouse blood.

### Ethics statement

All animal experiments were performed according to the methods approved by the Institutional Animal Care Committee of the University of Tokyo or the Animal Welfare Committee of PhoenixBio Co., Ltd. All animals received humane care according to the criteria outlined in the Guide for the Care and Use of Laboratory Animals prepared by the National Academy of Sciences and published by the National Institutes of Health.

### Animal study and specimen collection

1,2-DCP was dissolved in olive oil at 100 mg/mL with an administration volume of 5 mL/kg body weight (b.w.) for mice and rats. Groups of mice and rats were treated orally with 1,2-DCP (500 mg/kg b.w.) or vehicle control each day for four days. This dose of 1,2-DCP is higher than the carcinogenic dose of 1,2-DCP (250 mg/kg b.w.) in two-year studies using male B6C3F1 mice which were given an oral gavage treatment of 1,2-DCP for 103 weeks (5 days per week)^51^. No signs of mortality induced by 1,2-DCP was observed in any the animals during the present study.

Four hours after the latest administration of 1,2-DCP, bile specimens from each animal were collected by cannulation for one hour under the deep anaesthesia with urethane (1.25 g/kg b.w., i.p.). The bile duct was cannulated with a Teflon-coated tube (UT-03 type) (Unique medical Co., Ltd., Tokyo, Japan) for mice and PE tubing (PE8040) (Natsume seisakusyo Co., Ltd., Tokyo, Japan) for rats, respectively. Bile specimens were weighed, and bile volume was determined by assuming a specific gravity of 1.0 g/mL^52^. Just after bile collection, blood was taken immediately prior to euthanasia. Serum specimens were obtained as supernatants by centrifuging (3,000 × *g* at 4 °C for 10 min) the blood in a BD Microtainer with serum separator (BD biosciences, Tokyo, Japan). At necropsy, livers were excised and weighed, then rapidly frozen in liquid nitrogen. All specimens were stored at −80 °C until further processing.

For PXB mice, the similar experiment was performed with minor modifications at PhoenixBio Co., Ltd. In this experiment, bile specimens were collected by gall bladder cannulation from anesthetized PXB mice for 24 hours every 8 hours just after the oral administration of 1,2-DCP. At 8 and 16 hours, replenisher solution consisting of saline and 5% glucose solution 1:1 (v/v) was administered by subcutaneous injection (25 μL/g b.w.).

### Preparation of analytical sample

After thawing on ice, each specimen was preprocessed as follows prior to LC-MS/MS analysis. Each bile aliquot was deproteinized with four volumes of methanol, then the mixture was vortexed well for 2 min, and centrifuged at 15,000 × *g* for 10 min at 4 °C. The resulting supernatant was transferred to new glass vial, and then subjected to UPLC separation. For the liver tissue, approximately 100 mg of tissue was homogenized with 1 mL of water by Physcotron homogenizer (Microtec co., Ltd., Chiba, Japan), and then centrifuged at 3,000 × *g* for 10 min at 4 °C to remove debris and cell nuclei. The supernatant was mixed with four volumes of methanol, followed by the same procedure as described above. For serum samples, four volumes of methanol containing LTC4-d5 as internal control were added to the 100 μL of serum aliquot, followed by vortexing for 2 min. After the subsequent centrifugation (15,000 × *g* for 10 min at 4 °C), the supernatant was evaporated to dryness with a Centrifugal Evaporator EC-57C3 (SAKUMA, Tokyo, Japan). The dried extract was reconstituted in 100 μL of water/methanol (20:80), centrifuged (15,000 × *g* for 10 min at 4 °C), and the resulting supernatant was used for the next UPLC-MS/MS analysis.

### Untargeted metabolomics analysis using UPLC-ESI-MS/MS

Analysis of all samples was performed by a Thermo Scientific Q Exactive orbitrap LC-MS/MS System (Thermo Fisher Scientific K.K.) coupled with a DIONEX Ultimate 3000 Rapid Separation LC system (Thermo Fisher Scientific K.K.). A volume of 2 μL of sample was injected onto a Syncronis aQ columun (100 × 21 mm, 5 μm; Thermo Fisher Scientific K.K.) and then separated. Elution was performed using a gradient mobile phase (0–5 min: 0% B; 5–10 min: 0–90% B; 10–30 min: 90% B; 30–40 min: 0% B) of 0.1% formic acid in water (A) and 0.1% formic acid in acetonitrile (B) at a flow rate of 300 μL/min. The column and autosampler temperature were maintained at 40 °C and 10 °C, respectively. Ionization was performed by a heated electrospray ionization (ESI) probe in positive ion mode with the spray voltage in 3 kV and negative ion mode with the voltage in −2.5 kV, respectively. The S-lens level was set at 80. The capillary and vaporizer temperatures were set at 250 °C and 400 °C, respectively. Nitrogen sheath gas flow and auxiliary gas flow were set at 50 and 20 arbitrary units, respectively.

Detection was performed using a Q Exactive mass spectrometer controlled by Excalibur software (Thermo Fisher Scientific K.K.), and exact masses were calculated using Qualbrowser program (Thermo Fisher Scientific K.K.). Instrument calibration was performed 25 hours prior to every experiment with a direct infusion of calibration solution according to manufacturer’s instructions. Full MS scans were operated in full spectrum acquisition from m/z 100 to 800. The resolution was 70,000 FWHM at m/z 200 with a maximum injection time of 0.25 sec. The automatic gain control (AGC) was set at 1 × 10^6^. MS/MS data were acquired in the data-dependent MS^2^ (Top 5) mode with a resolution of 17,500 FWHM at m/z 200. The loop count was set at 5 and the AGC was 1 × 10^5^. For fragmentations, the HCD normalized collision energy was set to 30%, enabling the stepped collision energy (width 50%, 3 steps).

To detect differences between control and 1,2-DCP-administered conditions, we performed the differential analysis of profiling data using SIEVE software (Thermo Fisher Scientific K.K.), resulting in the generation of data set consisting of ion peaks (m/z, retention time; RT) and their respective intensities (peak areas). Peak detection and retention time correction were performed using the following parameters: mass range of 100–800 m/z, mass tolerance of 10 ppm, RT range of 0–40 min, and threshold for intensity of 50,000. To find the metabolites of 1,2-DCP, we focused on the peaks that were only detected in 1,2-DCP-administered group. The stability and reproducibility of the analytical result was confirmed by another data set derived from the samples that were collected on a different day, and then metabolite identification was carried out based on UPLC-MS/MS data; accurate mass, isotopic pattern, and fragment ions for target metabolites. To determine the relative levels of selected metabolites, we generated extracted ion chromatograms based on accurate mass, followed by peak integration using the Qualbrowser program.

### Statistical analysis

All statistical analyses were performed by using EXCEL 2013 (Microsoft, USA) with Statcel3 add-in software (OMS publishing Inc., Saitama, Japan). Different statistical tests were used for different experiments as described in Figure legends. All data of *in vivo* experiments were represented as means ± S.E.M. The significance of each value was determined when *P* value was less than 0.05 and 0.01.

*In vitro* reaction of 1,2-DCP with GSH, *in vitro* transport studies using ABCC2-expressing membrane vesicles, and other detailed methods are provided in Supplementary Information.

## Additional Information

**How to cite this article**: Toyoda, Y. *et al.* Halogenated hydrocarbon solvent-related cholangiocarcinoma risk: biliary excretion of glutathione conjugates of 1,2-dichloropropane evidenced by untargeted metabolomics analysis. *Sci. Rep.* **6**, 24586; doi: 10.1038/srep24586 (2016).

## Supplementary Material

Supplementary Information

## Figures and Tables

**Table 1:** Peak intensities of each metabolite in serum, bile, and liver of 1,2-DCP-administered SD rats.

GS-DCPs	Peak intensity (×10^8^)								
	No. 1	No. 2	No. 4	No. 6	No. 7	No. 8	No. 9	No. 10	No. 12
	No	Yes	No	No	Yes	Yes	Yes	No	Yes
Serum/mL	23.72 ± 4.47	N.D.	2.52 ± 0.51	N.D.	0.41 ± 0.05	0.47 ± 0.07	0.25 ± 0.05	2.98 ± 0.69	0.16 ± 0.03
Bile/mL	980.66 ± 211.07	23.61 ± 10.03	1.55 ± 0.69	253.73 ± 53.76	460.58 ± 135.93	362.67 ± 104.79	291.16 ± 104.23	3.80 ± 0.42	103.61 ± 27.00
Liver/g	175.36 ± 35.36	N.D.	N.D.	N.D.	46.02 ± 12.23	43.62 ± 11.20	7.81 ± 1.91	13.18 ± 4.14	7.58 ± 1.70

No. 10, mercapturate; No. 12, half mustard form of 1,2-DCP. N.D., not detected.

Data are expressed as the mean ± S.E.M. *n* = 9 (serum and bile), 6 (liver).
