# Supplementary material for: Halogenated hydrocarbon solvent-related cholangiocarcinoma risk: biliary excretion of glutathione conjugates of 1,2-dichloropropane evidenced by untargeted metabolomics analysis
Source: Sci Rep. 2016 Apr 18;6:24586. doi: 10.1038/srep24586 (PMC5263858; doi:10.1038/srep24586)
Supplement: Supplementary Information [file srep24586-s1.pdf]

SUPPLEMENTARY INFORMATION

Halogenated hydrocarbon solvent-related cholangiocarcinoma risk: biliary excretion of glutathione conjugates of 1,2-dichloropropane evidenced by untargeted metabolomics analysis

Yu Toyoda[*], Tappei Takada[*] & Hiroshi Suzuki

Department of Pharmacy, The University of Tokyo Hospital, 7-3-1 Hongo, Bunkyo-ku, Tokyo 113-8655, Japan.

[*]These authors contributed equally to this work. Correspondence and requests for materials should be addressed to T.T. (e-mail: tappei-tky@umin.ac.jp)

Supplementary Methods

Supplementary References

Supplementary Figures S1-S4

Supplementary Table S1

Supplementary Information

Supplementary Methods

In vitro reaction of 1,2-DCP with GSH and column separation

An aliquot of 1,2-DCP was added into 25-fold volumes of 100 mM potassium phosphate buffer pH 6.5 or 8.0 containing GSH (f.c. 6 mM). The mixture was vortexed well and incubated at indicated temperature for 12 hours. The production of GS-DCPs and the depletion of GSH in the resulting solution were confirmed by LC-MS/MS detection and colorimetric quantification of GSH as described in the following section, respectively. In order to remove GSH and replace the buffer, the resulting solution was separated by Sep-Pak Vac 35cc C18 Cartridges (Waters, Milford, MA) connected with Waters Vacuum manifold (Waters). In brief, the column was activated by 20 mL of methanol twice, and then treated with 25 mL of potassium phosphate buffer. Then, the reaction mixture was added into the column and eluted by water per 5 mL for 10 times. All fractions were collected and subjected to LC-MS/MS analysis. For transport experiments, GS-DCP rich fractions (fraction 7 to 10; total about 20 mL) were evaporated to dryness with a Centrifugal Evaporator EC-57C3 (SAKUMA, Tokyo, Japan). The dried extract was dissolved in water and was used.

GSH quantification by DTNB

The contents of GSH were measured spectrophotometrically with Ellman's reagent (DTNB) as descpribed elsewhere[1]. In brief, a stock solution of DTNB (0.5 mM DTNB in 0.1 M sodium phosphate buffer) was prepared and stored 4ºC before used. A 10 μL aliquot of each sample was diluted with four volumes of water, mixed

with the 150 μL of DTNB solution, and incubated for 10 min in room temperature. The resulting absorbance at 412 nm was read by Varioskan flash microplate reader (Thermo Fisher Scientific K.K.). The GSH solutions of known concentration were used for the preparation of a standard curve.

In vitro transport studies using ABCC2-expressing membrane vesicles

Transport experiments of GS-DCPs into human ABCC2-expressing and control vesicles were performed as described in a previous report[2] with some modifications. In brief, ABCC2-expressing or control plasma membrane vesicles (175 μg of protein) were incubated with GS-DCPs-containing solutions in the presence of 5 mM ATP or AMP (ATP(-)) in 250 μL of the transport buffer (0.25 M sucrose and 10 mM Tris-HCl, pH 7.4, 10 mM $MgCl_2$, 100 mM creatine phosphate, 100 mg/mL creatine phosphokinase type I and 30 μL of GS-DCPs fraction) for 10 min at 37ºC. After the incubation, the reaction mixture was mixed with 750 μL of ice-cold stop buffer (2 mM EDTA, 0.25 M sucrose, 0.1 M NaCl, and 10 mM Tris-HCl, pH 7.4) and then the resulting solution was rapidly filtered by 0.45 μm HVLP Durapore hydrophilic PVDF membrane filter (Millipore Corporation, Billerica, MA). After washing with 5 mL of ice-cold stop buffer twice, membrane vesicles on the filter was dissolved in the methanol containing LTC_4-d5 (2.5 ng/mL) as an internal control. The extract was evaporated to dryness with a Centrifugal Evaporator EC-57C3, and then resolved in 100 μL of 80% MeOH. The resulting solution was subjected to LC-MS/MS analysis in order to determine the levels of GS-DCPs that were incorporated into the vesicles. Transport activity was calculated as an incorporated clearance (mL/mg protein/min = incorporated level of GS-DCP (intensity/mg protein/min) / GS-DCP level in incubation

mixture (intensity/mL)). ATP-dependent transport was calculated by subtracting the transport activity in the absence of ATP from activity in the presence of 5 mM ATP.

Supplementary References

1 Riddles, P.W., Blakeley, R.L. & Zerner, B. Ellman's reagent: 5,5'-dithiobis(2-nitrobenzoic acid)--a reexamination. *Anal. Biochem.* **94**, 75-81 (1979).

2 Yoshikado, T. *et al.* Ticlopidine, a cholestatic liver injury-inducible drug, causes dysfunction of bile formation via diminished biliary secretion of phospholipids: involvement of biliary-excreted glutathione-conjugated ticlopidine metabolites. *Mol. Pharmacol.* **83**, 552-562 (2013).

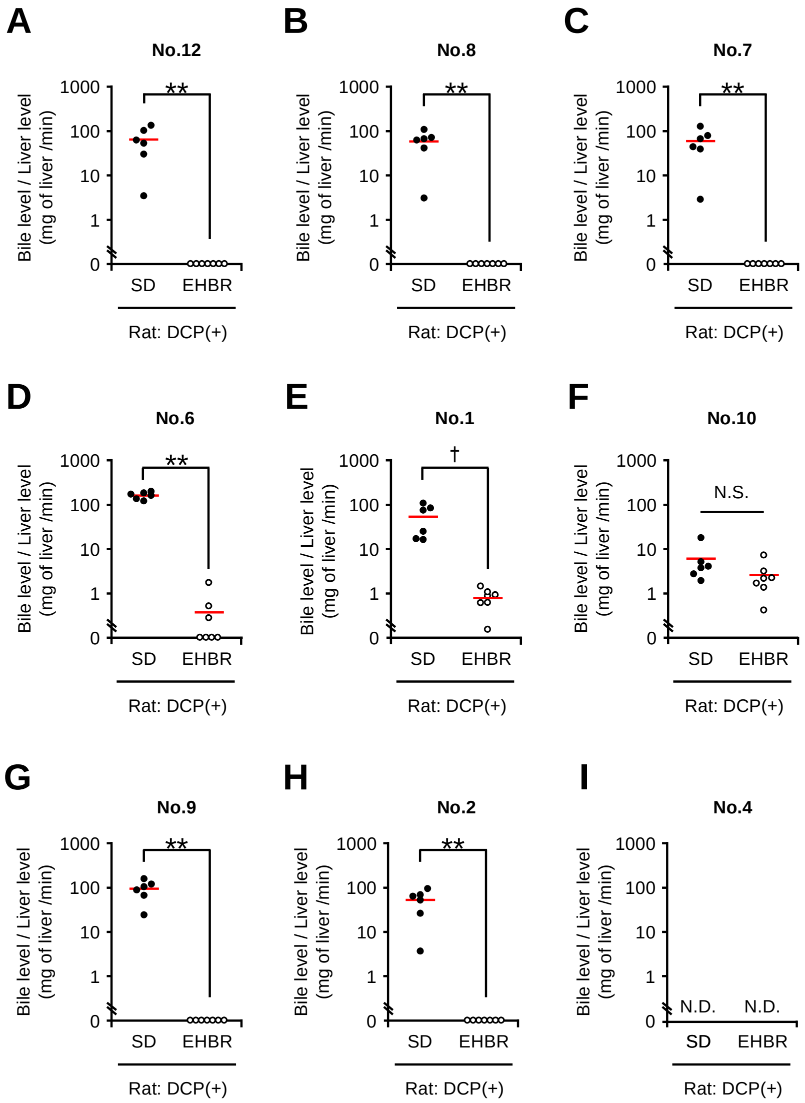

Supplementary Figure S1. Comparison of the ratio of biliary level/liver level of each 1,2-DCP metabolite between 1,2-DCP-administered SD rats and EHBRs. The ratio of biliary level/liver level, biliary clearance of each 1,2-DCP metabolite (mg of liver/min), was calculated by dividing the intensity of each metabolite in bile per unit time (intensity/min) by that in the liver per unit weight (intensity/mg of liver). The red bars indicate the mean of value in each group. Statistical analyses for significant differences were performed according to a parametric Student's *t* test (†, $P<0.05$) or a nonparametric, Mann-Whitney *U* test (**, $P<0.01$). N.D. not determined.

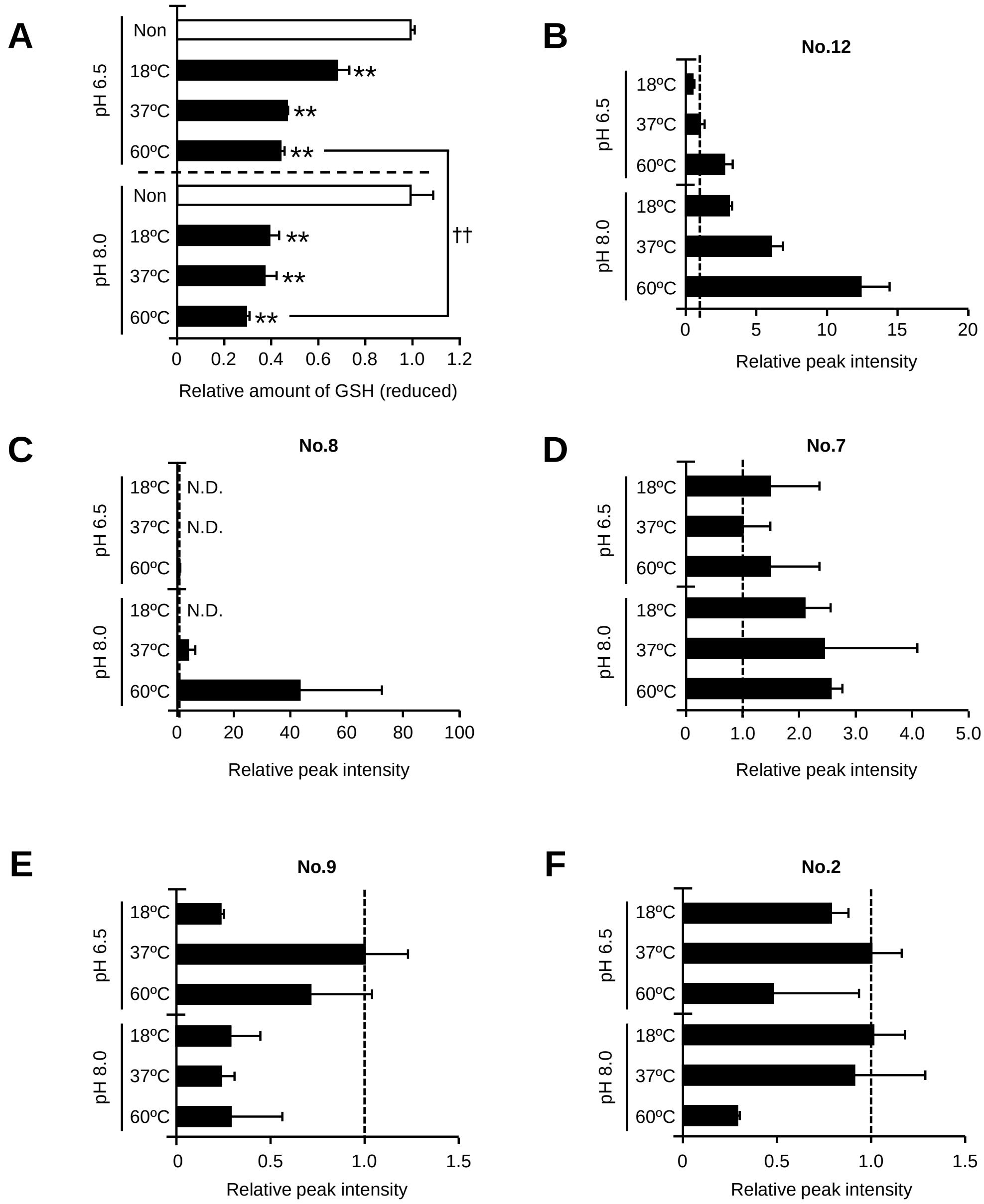

Supplementary Figure S2. ***In vitro*** **reaction of 1,2-DCP with GSH.** 1,2-DCP and GSH were mixed well and incubated at indicated temperature for 12 hours, then the GSH and metabolite levels of each metabolite in the resulting solution were analysed. (A) Relative amount of GSH. The level of remaining GSH in each experimental condition was normalized by the initial amount of GSH (6 mM in incubation mixture). Data were expressed as means ± S.D. n = 3. Statistical analyses for significant differences were performed according to Student's t test (††, P<0.01) and Bartlett's

test, followed by Williams' test (**, P<0.01 vs. control in each buffer condition). (B-F) Relative levels of each metabolite. (B), No.12; (C), No.8; (D), No.7; (E), No.9; (F), No.2. All metabolites of 1,2-DCP detected in this *in vitro* experiment were glutathione conjugates. On the other hand, No.6, No.1, No.10 and No.4 were not detected. Because, at least, No.6, No.1, and No.10 would be enzymatically-produced metabolites, *in vitro* incubation could not have resulted in the production of these compounds. Vertical dashed lines indicate the normalized value. Data were expressed as means ± S.D. n = 3.

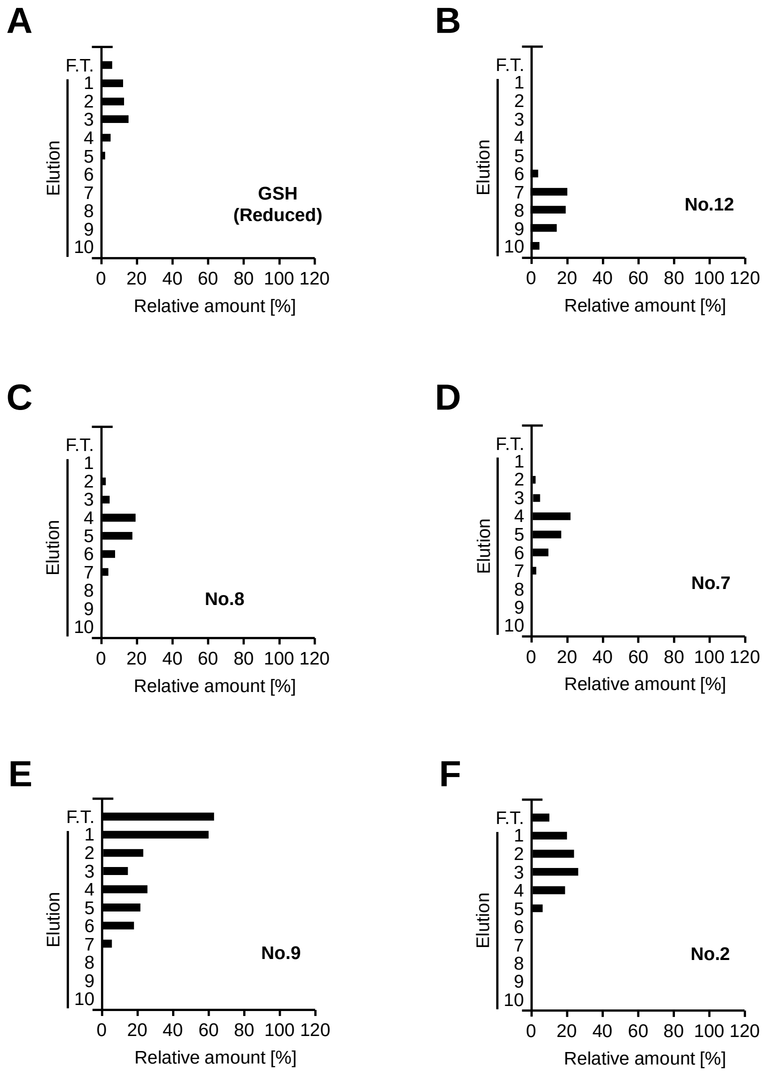

Supplementary Figure S3. Column separation of *in vitro* reaction mixture containing GS-DCPs. The resulting solution of *in vitro* reaction of 1,2-DCP with GSH, input solution, was separated by Sep-Pak Vac 35cc C18 Cartridges, then each fraction was subjected to LC-MS/MS analysis. Relative amount of each 1,2-DCP metabolite and GSH in each fraction was calculated by dividing the intensity of each fraction by that of input solution. F.T., flow through. (A), GSH; (B), No.12; (C), No.8; (D), No.7; (E), No.9; (F), No.2.

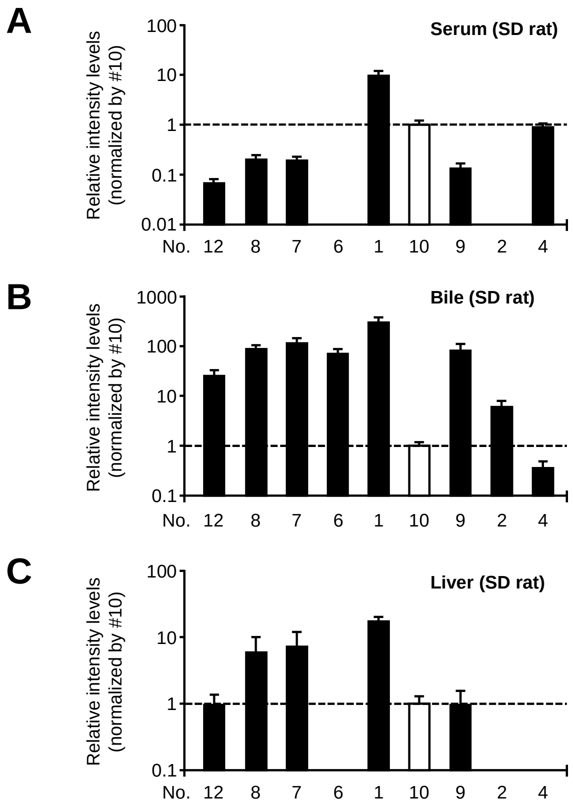

Supplementary Figure S4. Relative intensity levels of each 1,2-DCP metabolite. Total intensities of each metabolite in (A) serum, (B) bile, (C) liver of 1,2-DCP-administered SD rat were normalized by those of No.10. No.1, No.10 and No.4 may be promising candidates for serum biomarker. Data are expressed as the mean ± S.E.M. n = 9 (A and B), 6 (C).

Supplementary Table S1. List of candidates of 1,2-DCP metabolite in bile of 1,2-DCP-administered rodents. Based on the results of untargeted metabolomics analyses followed by differential analyses, using bile specimens obtained from C57BL/6J mice treated with or without 1,2-DCP, 13 compounds of interest were selected. These compounds were not detected in control (vehicle) groups. We have described two metabolic pathways of 1,2-DCP in **Fig. 1** including nine compounds written in black letters. In UPLC separation for the differential analyses and acquisition of structure information, acetonitrile was used as mobile phase B (LC#1). For other experiments, 0.1% formic acid in acetonitrile was used as mobile phase B (LC#2).

No.	Retention time (min) LC#1	LC#2	m/z (positive)	m/z (negative)	Formula
1	1.34	1.19	180.0687	178.0549	C6H13O3NS
2	1.37	0.91	382.1276	380.1145	C13H23O8N3S
3[a]	1.37	1.25	194.0480	192.0342	C6H11O4NS
4	1.76	1.48	123.0473	N.D.	C4H10O2S
5[b]	1.92	-	290.1340	288.1208	C11H19O6N3
6	2.23	1.82	237.0901	N.D.	C8H16O4N2S
7	3.88	3.13	364.1170	362.1040	C13H21O7N3S
8	4.05	3.27	366.1322	364.1192	C13H23O7N3S
9	4.64	3.97	380.1118	378.0987	C13H21O8N3S
10	9.16	7.37	222.0791	220.0655	C8H16O4NS
11[b]	11.24	-	408.1432	406.1301	C15H25O8N3S
12	13.36	8.18	382.0831	380.0702	C13H20O6N3ClS
13[b]	14.71	-	248.0949	246.0813	C10H18O4NS

N.D., not detected

[a] Product ion spectrum was not obtained probably due to the low intensity.

[b] We could not determine whether these compounds were derived from 1,2-DCP or not.
